# *dlk1*促进肺鳞癌细胞增殖的相关分子机理研究

**DOI:** 10.3779/j.issn.1009-3419.2010.10.12

**Published:** 2010-10-20

**Authors:** 宇 刘, 金晶 谭, 琳 李, 硕 李, 霜梅 邹, 莹 张, 晓静 张, 兵 凌, 迺珺 韩, 素萍 郭, 燕宁 高

**Affiliations:** 1 100021 北京，北京协和医学院，中国医学科学院，肿瘤医院肿瘤研究所，癌发生及预防分子机理北京市重点实验室/病因及癌变研究室 Department of Etiology and Carcinogenesis, Peking Union Medical College & Chinese Academy of Medical Sciences, Beijing 100021, China; 2 100021 北京，北京协和医学院，中国医学科学院，肿瘤医院肿瘤研究所，癌发生及预防分子机理北京市重点实验室/病理科 Department of Pathology, Cancer Institute/Hospital, Peking Union Medical College & Chinese Academy of Medical Sciences, Beijing 100021, China

**Keywords:** *dlk1*, 肺癌, 细胞增殖, CyclinB1, *dlk1*, Lung neoplasms, Proliferation, CyclinB1

## Abstract

**背景与目的:**

印记基因*dlk1*因在多种肿瘤组织中出现异常表达而受到研究者越来越多的关注，但*dlk1*基因与肺癌的关系尚无报道。本研究首先在肺癌组织中检测了*dlk1*基因的表达，并进一步利用肺癌细胞系H520对*dlk1*基因促进细胞增殖的分子机制进行了初步研究。

**方法:**

首先，采用RT-PCR在30对非小细胞肺癌肿瘤及其配对癌旁组织中检测*dlk1*基因的表达。然后，克隆人源*dlk1*基因，转染并筛选出稳定表达*dlk1*基因的肺癌细胞。最后，利用CCK8法研究*dlk1*基因对细胞增殖能力的影响，并用Western blot技术分析细胞周期蛋白CyclinB1的表达。

**结果:**

RTPCR结果显示，*dlk1*基因在36.7%的非小细胞肺癌肿瘤组织中表达水平高于癌旁肺组织。在成功获得了稳定表达外源性*dlk1*基因的肺癌细胞H520-*dlk1*的基础上，CCK8实验及平板集落实验显示，稳定转染*dlk1*可以明显促进肺鳞癌细胞H520的增殖能力（*P* < 0.05）。同时，稳定表达DLK1蛋白可以上调细胞周期蛋白CyclinB1的表达水平（*P* < 0.05）。

**结论:**

*dlk1*在非小细胞肺癌中存在异常高表达，它可以通过上调细胞周期蛋白CyclinB1的表达，促进肺鳞癌细胞H520的增殖。提示*dlk1*基因的异常表达可能在肺癌的发生演进中发挥作用。

## Abstract (en)

**Background and objective:**

The imprinted gene *dlk1* has been recognized as a cancer related gene since it aberrantly expressed in a series of cancer tissues, but its role in lung cancer is still unknown. The aim of this study is to examine *dlk1*'s expression in non-small cell lung cancers (NSCLCs) and investigate the molecular mechanism by which *dlk1* could accelerate the proliferation of the cells in lung cancer cell lines (H520).

**Methods:**

The relative expression of *dlk1* among 30 NSCLC specimens and their adjacent normal lung tissues were analyzed by RT-PCR. A cell model that stably expressed exogenous *dlk1* was established following that the *dlk1* gene was cloned into a eukaryotic expression vector and then transfected into the lung cancer cells H520. CCK8 analysis and colony forming assay were employed to investigate the effect of *dlk1* on cell proliferation. The expression of CyclinB1 was detected by Western blot.

**Results:**

*dlk1* aberrantly expressed in 36.7% (11/30) of the tumor tissues of NSCLC compared with their adjacent cancer lung tissues. CCK8 analysis showed that overexpression of *dlk1* could promote the proliferation of H520 cells (*P* < 0.05) and the results was further confirmed by colony forming assay. Western blot analysis found that over expression of *dlk1* could up-regulate the expression of CyclinB1 (*P* < 0.05).

**Conclusion:**

*dlk1* aberrantly expressed in NSCLCs. The Overexpression of *dlk1* could accelerate the proliferation of lung cancer cells H520 *in vitro*, probably through up-regulating the expression of cell cycle protein CyclinB1.

肺癌严重危害人类健康与生命。2008年美国肺癌新增病例数及死亡病例数分列各种肿瘤的第二位及第一位^[1]^。中国国家卫生部公布的资料^[2]^显示，在过去30年间肺癌死亡率上升了465%，已经成为我国恶性肿瘤死亡的首位原因。肺癌的发生是基因与环境相互作用的结果，其中涉及到多种癌基因的激活与抑癌基因的失活^[3, 4]^。本实验室利用基因芯片技术对82例肺鳞癌患者的肿瘤组织与配对癌旁组织的mRNA表达谱分析显示，*dlk1*基因在肺鳞癌中表达水平显著高于癌旁正常组织（尚未发表的资料），提示*dlk1*基因可能在肺鳞癌的发生演进过程中起促进作用，值得深入研究。

*dlk1*基因别名FA1、ZOG、Pref-1，定位于人14号染色体长臂14q32，编码含383个氨基酸的蛋白质DLK1，属父源性印迹基因。DLK1蛋白由N端的信号肽，6个EGF结构域，一个跨膜结构域及C端的胞内肽段组成，属于表皮生长因子类家族蛋白，与Notch/Delta/Serrate蛋白具有一定的同源性。其蛋白结构与其同源蛋白DLL1相比，缺少N端的DSL结构域^[5]^。*dlk1*基因在大部分成体动物组织中处于沉默状态。根据GeneCards数据库（http://www.genecards.org/）中基因表达序列分析（Serial Analysis of Gene Expression）数据预测*dlk1*基因在人不同组织中的表达，结果表明，该基因在胎盘组织高表达，而在大部分正常组织中均不表达。最近的研究发现，*dlk1*基因在多种肿瘤中出现异常表达，如在脑胶质瘤、肝癌组织中表达水平均高于正常组织^[6, 7]^；且*dlk1*在肝癌中的高表达与肝癌病人预后不良相关^[8]^。而在肾癌中*dlk1*基因较正常组织低表达^[9]^。目前，*dlk1*基因与肺鳞癌的关系尚未见报道。

## 1 材料与方法

### 1.1 材料

本研究入组病例均为中国医学科学院肿瘤医院胸外科收治的肺癌患者，术前均未接受放射治疗或化学治疗。全部患者均接受了规范的肺癌根治手术及区域淋巴结清扫治疗。组织病理学诊断依据国际抗癌联盟（International Union Against Cancer, UICC）2002年标准。人肺鳞癌细胞株H520源自美国ATCC；真核表达载体pcDNA3.1-Myc（His）购自Invitrogen公司；DLK1抗体购自Abcam公司；CyclinB1抗体购自Santa Cruz公司；Cell Counting Kit-8（CCK8）试剂盒购自日本同仁化学研究所。

### 1.2 方法

#### 1.2.1 细胞培养

肺鳞癌细胞H520在含10%胎牛血清的RPMI-1640培养基中，于37 ℃、5%CO_2_条件下常规贴壁培养。待细胞生长汇合度达80%左右时，以含0.2%EDTA的0.25%胰酶消化细胞，进行常规传代。

#### 1.2.2 反转录-聚合酶链式反应（RT-PCR）

提取组织或细胞总RNA，取1 µg总RNA按照Invitrogen反转录试剂盒进行逆转录反应。取1 µL反转录后的cDNA为模板进行PCR反应。*dlk1*引物F：5'-AAGGACTGCCAGAAAAAGGAC-3'，R：5'-GCAGAAATTGCCTGAGAAGC-3'；产物长度138 bp。18s引物F：5'-GAAACGGCTACCACATCC-3'，R：5'-ACCAGACTTGCCCTCCA-3'，产物长度167 bp，退火温度均为60 ℃。

#### 1.2.3 *dlk1*基因的克隆和真核表达质粒的构建

根据NCBI数据库中*dlk1*基因mRNA序列信息（Accession Number: NM_003836.4）设计克隆引物，在上游引物中含有*Nhe*Ⅰ酶切位点，下游引物含有*Hin*dⅢ酶切位点。PCR扩增*dlk1*基因全长开放读码框（ORF）。产物经纯化、酶切后，连进入真核表达载体pcDNA3.1/mycHis(-)。连接产物转化感受态细胞，并筛选获得成功转化的阳性克隆进行后续鉴定。*dlk1*克隆引物F：5'-CCCAAGCTTGGGGATCTCCTCGTCGCCG-3'，R：5'-CCCAAGCTTGGGGATCTCCTCGTCGCCG-3'；产物长度1 257 bp，采用两步法进行扩增，退火及延伸温度均为72 ℃。

#### 1.2.4 脂质体介导的细胞转染

按Lipofectamine^TM^ 2000产品说明书操作。待细胞生长至70%-90%融合时进行转染，将混匀的质粒与脂质体加入细胞培养液中，于37 ℃、5%CO_2_条件下培养。并于4 h-6 h后更换新鲜培养基。在转染质粒24 h后，培养基中加入G418（500 µg/mL）筛选稳定表达外源基因的细胞，分别命名为H520-dlk1及H520- pcdb。

#### 1.2.5 细胞生长曲线的绘制

参照生产商说明书进行实验。将一定数量的细胞种于96孔板中，37 ℃常规培养，并记为第0天。测定细胞活性时，按照100 µL培养基加入10 µL CCK8溶液的比例稀释CCK8；更换细胞培养基后，将稀释好的CCK8溶液加入孔板中；37 ℃培养2 h，并于450 nm波长下测定吸光度值。

#### 1.2.6 Western blot分析

采用RIPA裂解缓冲液提取细胞总蛋白，BCA Protein Assay Kit对蛋白进行定量。取80 µg变性后的蛋白进行实验。所用抗DLK1抗体及抗CyclinB1的抗体稀释度分别为1:100及1:1 000，并用ECL发光试剂检测目的蛋白表达。

### 1.3 统计学分析

采用R统计分析平台对实验结果进行分析，以*t*检验进行统计学分析，以*P* < 0.05为有统计学差异。

## 2 结果

### 2.1 *dlk1*基因在肺癌组织中的表达

通过RT-PCR方法在30例非小细胞肺癌（其中鳞癌16例，腺癌14例）患者的组织及其配对癌旁正常组织中检测了*dlk1*基因mRNA的表达情况。结果显示，*dlk1*基因在36.7%（11/30）的非小细胞肺癌组织中较癌旁组织高表达（图 1，差异表达倍数≥2.0）。

**1:**
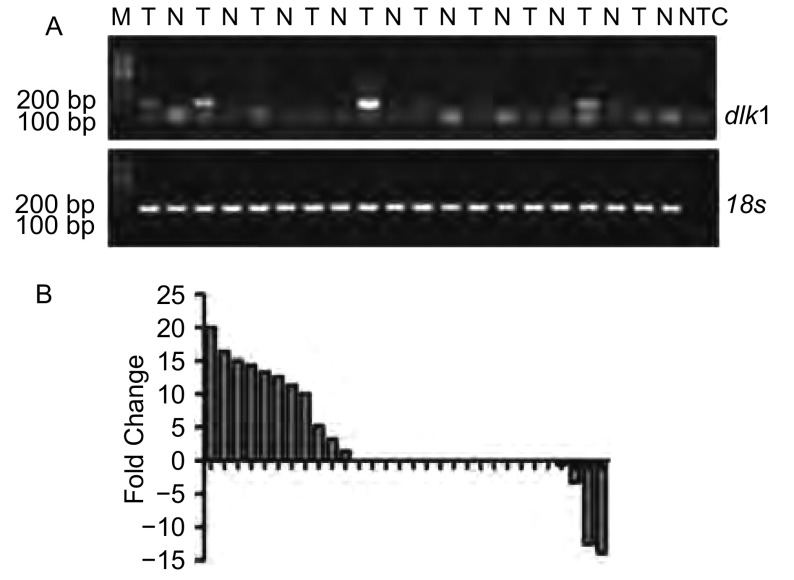
*dlk1*基因在30例非小细胞肺癌组织及其癌旁正常组织中的表达。A：*dlk1*基因在非小细胞肺癌组织中表达情况的琼脂糖凝胶电泳示意图，18s为内参对照基因；B：*dlk1*基因在30例非小细胞肺癌组织与其配对癌旁正常组织表达情况的比较。 Expression pattern of *dlk1* in 30 non-small cell lung cancer (NSCLC) specimens and the paired adjacent normal lung tissues. A: Representative results of RT-PCR of *dlk1* from NSCLC (T) and their adjacent non-cancerous lungs (N), where 18s was employed as internal control; B: A histogram represents the relative expression of *dlk1* among 30 NSCLC specimens and their adjacent normal lung tissues.

### 2.2 含正确*dlk1*基因序列的真核表达载体的获得

随机挑取不同的5个菌落，小规模制备质粒DNA后，经酶切电泳鉴定，1-3号质粒中插入了目的基因*dlk1*（酶切产物长度与PCR产物长度一致，图 2）。取这3个细菌克隆提取质粒，经测序鉴定显示，2号质粒与NCBI数据库中目的序列一致，用作后续实验研究。

**2:**
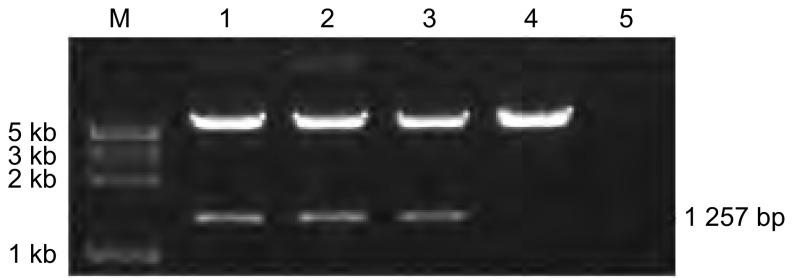
不同菌落小规模制备质粒DNA后经限制性核酸内切酶双酶切后电泳图。M：DNA Lader，1-5为质粒编号。插入的目的基因长度为1 257 bp，真核表达载体pcDNA3.1长度为5.5 kb。 Electrophoretogram of 5 different plasmids digested by restriction endonuclease. M: DNA Lader, 1-5: plasmid ID. The inserted fragment of target gene dlk1 was 1 257 bp and the vector pcDNA3.1 was 5.5 kb in length.

### 2.3 稳定表达外源性*dlk1*基因的H520细胞的获得与筛选

分别收集作为空白对照的亲本H520细胞、H520-pcdb和H520-dlk1细胞，提取细胞RNA及蛋白质。分别进行RTPCR（图 3A）及Western blot（图 3B）分析。结果显示，H520-dlk1细胞中在RNA及蛋白水平均可检测到外源性*dlk1*基因的表达，而在空白H520细胞及空载体H520-pcdb细胞中，均未检测到*dlk1*，表明已经成功筛选获得稳定表达外源性*dlk1*基因的H520细胞，用于后续研究。

**3:**
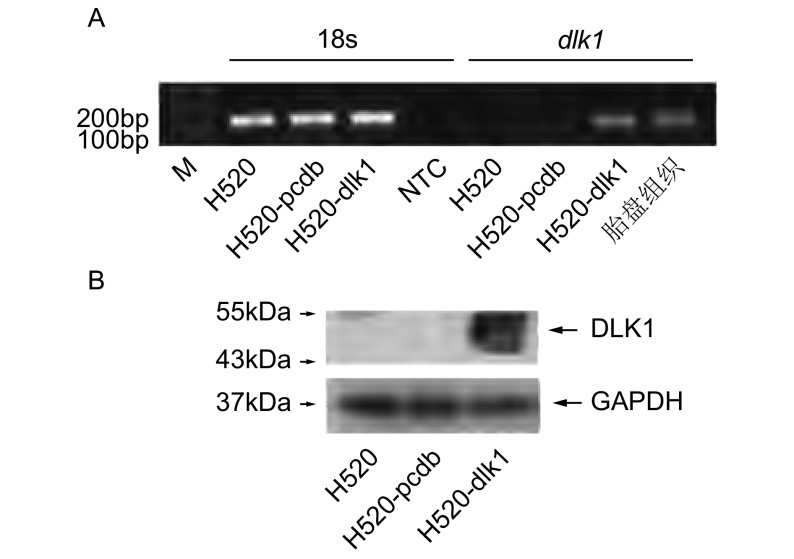
外源性*dlk1*基因在H520细胞中的表达鉴定。A：RT-PCR检测*dlk1*基因在空白H520细胞、H520-pcdb细胞及H520-dlk1细胞中的表达，M：Marker，NTC：无模板的PCR阴性对照，胎盘组织cDNA为阳性对照，18s为内参对照基因。B：Western Blot检测DLK1蛋白在上述3种细胞中的表达，GAPDH为内参对照。 The expression of *dlk1* in H520 cell was examined by both RTPCR and Western blot analysis. A: RT-PCR analysis of *dlk1* expression in blank H520, H520-pcdb and H520-dlk1 cells. M: Marker, NTC: none template control, placenta cDNA was used as positive control. B: Western blot analysis of *dlk1* expression.

### 2.4 *dlk1*基因过表达对肺鳞癌细胞增殖的影响及其调控的分子机制

以CCK8方法绘制细胞生长曲线，观察稳定表达*dlk1*基因对细胞增殖能力的影响，并采用平板集落形成实验对这一现象进行验证。如图 4A所示，稳定表达*dlk1*基因的H520-dlk1细胞与转染空载体的H520-pcdb细胞及空白的H520细胞相比细胞生长速度明显加快，而空载组H520-pcdb与空白组H520细胞相比细胞生长速度则无明显差别。平板集落实验结果（图 4B）同样发现稳定表达*dlk1*基因可以促进H520细胞的集落形成能力。同样条件下，H520-dlk1细胞的集落形成数量与H520-pcdb组及H520空白组相比均有明显增加，结果有统计学意义。而空载体组与空白组细胞间无统计学差异。

**4:**
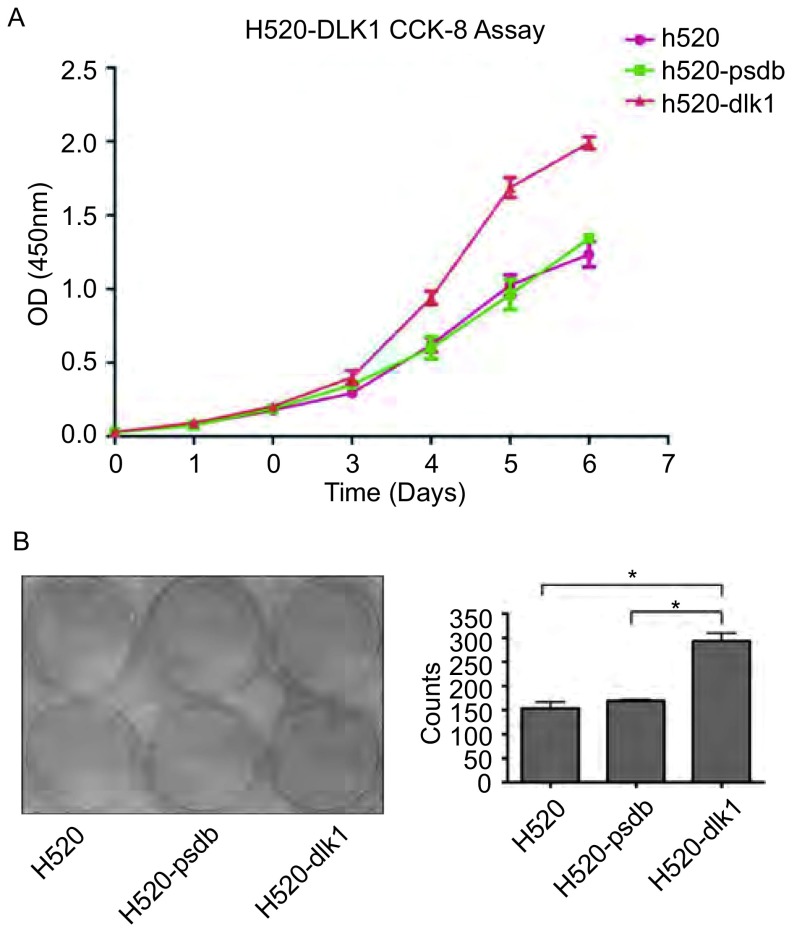
稳定表达*dlk1*基因对细胞体外增殖能力的影响。A：以CCK8绘制细胞生长曲线。横坐标为时间（单位天），纵坐标为CCK8在450 nm处的OD值。B：细胞集落形成实验结果及计数柱形图（^*^t-test，*P* < 0.05）。 *dlk1* accelerates cell proliferation *in vitro*. A: CCK8 analysis based cell growth curve. X axis represents time in days while Y axis is the absorption of CCK8 in 450 nm. B: Clone forming assay representations and histogram of the clone counts (^*^t-test, *P* < 0.05).

### 2.5 *dlk1*基因过表达对细胞周期蛋白CyclinB1表达水平的影响

利用Western blot技术，对细胞中CyclinB1的表达情况进行了分析。结果发现（图 5），与空载组H520-pcdb及空白组H520细胞相比，稳定表达DLK1蛋白的H520- dlk1细胞中CyclinB1的表达明显上调，结果具有统计学意义。而空载组与空白组间无明显差异。

**5:**
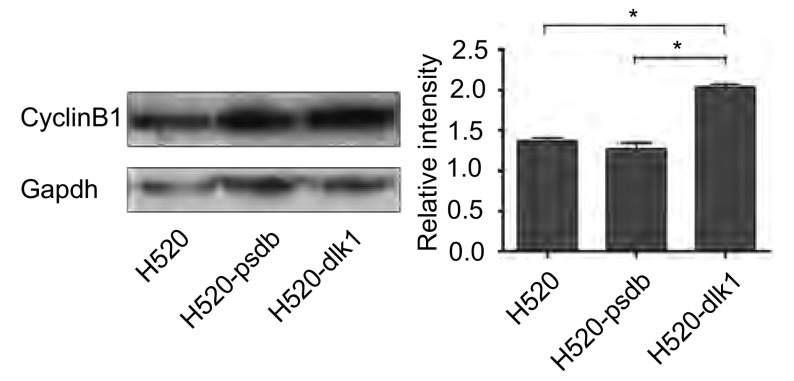
稳定表达*dlk1*基因对细胞中CyclinB1表达水平的影响。柱形图中纵坐标为以Gapdh为内参对照基因矫正后的CyclinB1相对表达量（^*^t-test，*P* < 0.05）。 *dlk1* induces CyclinB1 expression in H520 cells. The histogram represents the relative expression of CyclinB1 adjusted by housekeeping gene Gapdh (^*^t-test, *P* < 0.05).

## 3 讨论

本实验室前期针对82例肺鳞癌患者的肿瘤及其对照组织的mRNA表达谱分析发现，*dlk1*基因在肺癌组织中较癌旁正常组织高表达（尚未发表的资料）。本项研究采用另外30例非小细胞肺癌肿瘤及配对癌旁组织证实了*dlk1*基因确实在非小细胞肺癌中存在异常高表达。此外，本项研究的预实验显示，*dlk1*基因在一系列非小细胞肺癌细胞系中表达。为进一步探讨*dlk1*基因的分子功能，我们选择了不表达内源性*dlk1*基因的肺癌细胞系H520作为模型，进行后续体外实验研究。

细胞周期调控失效、细胞无限增殖是肿瘤细胞的特征之一。已有研究发现，肿瘤细胞中一系列细胞周期相关蛋白出现异常高表达，其中包括CyclinD1^[10, 11]^、CyclinE^[12, 13]^、CDC25A^[14]^等。而细胞的分化与增殖是一组对立的关系。在生物体内，处于终末分化状态、行使特定功能的细胞往往不具有增殖的能力。相反，保持旺盛增殖能力的细胞均为干细胞，并不具有特定的细胞行为。*dlk1*参与多种细胞分化过程的调节，其功能必然与细胞的增殖有某种关系。Huang等^[7]^报道，过表达DLK1蛋白可以导致肝癌细胞系SMMC-7721增殖加快，而利用siRNA抑制内源性*dlk1*基因表达可以降低细胞体外集落形成率，并抑制细胞在裸鼠体内的成瘤性。Yin等^[6]^在对脑胶质瘤的研究中得到了同样的结果，并且发现*dlk1*基因高表达可引起CyclinD1、CDK2、E2F1等蛋白表达上调，并认为这可能是其引起的细胞增殖加快的可能原因之一。Kim^[15]^与Ruiz-Hidalgo^[16]^等研究发现*dlk1*基因可以引起ERK蛋白磷酸化，进而激活MAPK信号通路，并且这种激活作用存在剂量、时间依赖关系。考虑到肿瘤异质性以及肿瘤微环境对肿瘤的影响，*dlk1*基因在不同肿瘤中的作用及分子机制可能存在差别，而其在肺癌发生发展中的作用尚不清楚。我们利用稳定表达*dlk1*基因的H520肺癌细胞模型研究*dlk1*基因与肺癌细胞增殖之间的关系。结果显示，稳定表达*dlk1*基因可以显著增加细胞的增殖速度，并可以增加细胞的集落形成能力（图 4）。这与文献中的报道是一致的。

进一步的Western blot结果则显示，稳定表达*dlk1*基因后，可以引起CyclinB1表达的上调（图 5）。Cyclin家族蛋白是一类细胞周期相关蛋白，不同类型的Cyclin蛋白在细胞周期的不同阶段特异性表达，从而调控细胞周期的有序、正常进行。研究表明，CyclinD在整个细胞周期中均有较高表达，其与CDK4/5结合，在细胞周期G_1_/S期转换过程中起到调控作用。而CyclinB在细胞进入S期后开始出现表达，其表达水平的峰值出现在细胞由G_2_期进入M期过程中。CyclinD与CyclinB1分别在细胞周期的G_1_/S及G_2_/M检验点起到调控作用。以往的报道均集中于*dlk1*基因高表达引起的CyclinD表达上调及细胞由G_1_期进入S期的增加，却没有对细胞周期中其他蛋白表达水平变化的相关报道。我们则发现*dlk1*基因高表达可以导致CyclinB1表达水平上调，提示*dlk1*基因可能通过作用于多个细胞周期相关蛋白，在多个细胞周期检验点中调节细胞的增殖能力，这为*dlk1*基因引起的细胞增殖能力增强的分子机制提供了新的线索。

在获得上述结果的基础上，我们还应注意到，虽然DLK1属于表皮生长因子类家族蛋白，但其与DLL1相比缺少DSL结构域，而这个结构域在激活下游信号通路中起着重要作用，提示DLK1蛋白可能通过其他途径向胞内传递信号，调节细胞增殖。因此，准确鉴定DLK1的相互作用蛋白及其参与调控的胞内信号通路具有重要意义，需要进一步的实验进行研究。
